# Rapid detection of *FadA* in *Fusobacterium nucleatum* using the quantitative LAMP colorimetric phenol red method in stool samples from colorectal cancer patients

**DOI:** 10.1038/s41598-024-62846-x

**Published:** 2024-06-14

**Authors:** Abdulrahman A. Zuraik, Yaman Daboul, M. Ayman Awama, Haitham Yazigi, Moh’d Azzam Kayasseh, Michael Georges

**Affiliations:** 1 Department of Biochemistry and Microbiology, Faculty of Pharmacy, Tishreen University, Lattakia, Syria; 2 School of Biological Sciences, Queens University Belfast, Belfast, UK; 3 Department of Laboratory Medicine/Faculty of Medicine, Tishreen University, Tishreen University Hospital, Lattakia, Syria; 4 Dr. Kayasseh Medical Clinic, Dr. Sulaiman Al-Habib Medical Group, DHCC, Dubai, UAE; 5 Department of Oncology, Faculty of Medicine, Tishreen University, Tishreen University Hospital, Lattakia, Syria

**Keywords:** QLAMP, Phenol red, *Fusobacterium nucleatum*, *FadA*, Colorectal cancer (CRC), Biotechnology, Cancer, Computational biology and bioinformatics, Developmental biology, Microbiology, Molecular biology, Gastroenterology, Medical research, Molecular medicine, Oncology

## Abstract

The study aimed to develop a quantitative colorimetric loop-mediated isothermal amplification technique using the phenol red indicator (QLAMP-PhR) for detecting *Fusobacterium nucleatum* (*Fn*) levels in colorectal cancer (CRC) patients and healthy individuals. QLAMP-PhR assays were conducted on 251 stool samples specific for the *Fn FadA* gene. Six primers were synthesized and utilized with master mix reagents, and a phenol red indicator was employed to enhance the QLAMP-PhR technique. A standard quantitative analysis curve was generated using a logarithmic function (absorbance vs. concentration) by serially diluting the copy number of genomic DNA templates (*Fn* ATCC25586). The CRC group exhibited a significantly higher abundance of *Fn* compared to the healthy control group (*P* < 0.001). These findings suggest that the QLAMP-PhR technique effectively identifies *Fn* specifically by its gene for the key virulence factor *FadA*. Additionally, ideas for developing a real-time QLAMP-PhR test were presented. Compared to the traditional polymerase chain reaction (PCR) technique, QLAMP-PhR offers several advantages including rapidity, simplicity, specificity, sensitivity, and cost-effectiveness method that can quantitatively screen for *Fn* presence in normal populations. The QLAMP-PhR method represents a sensitive and specific amplification assay for the rapid detection of the *Fn* pathogen. To the best of our knowledge, this study is the first to report the application of QLAMP-PhR for detecting *FadA* in *Fn*.

## Introduction

In the Middle East region, colorectal cancer (CRC) ranks as the third most common malignancy, with incidence and mortality rates similar to those in Asia and Western countries^1^. The gut microbiota has been increasingly associated with CRC, highlighting its significant role in CRC development^2,3^. A key player in this context is *Fusobacterium nucleatum* (*F. nucleatum*), an anaerobic, gram-negative bacterium present in both oral and gut microbiota^4^. Studies have linked *F. nucleatum* to human CRC, as confirmed by whole-genome metagenome analyses, transcriptome, and bacterial 16S ribosomal RNA sequencing^5–7^. The study focuses on the association between *F. nucleatum* and CRC. Through the development of the loop-mediated isothermal amplification (LAMP) method, the study aims to offer a rapid and cost-effective approach to detect and identify *F. nucleatum* as a potential diagnostic and prognostic marker for early CRC detection, facilitating precise monitoring of CRC cases and potentially enhancing early intervention strategies. Evidence suggests that *F. nucleatum* is abundant in tumor tissues and stool samples of CRC patients, indicating its role in CRC progression^8,9^. They are proposed as a risk factor for CRC progression^10–13^. For instance, a Chinese study analyzing fecal microbial communities in healthy individuals and CRC patients demonstrated a significant increase in *F. nucleatum* in the CRC cohort compared to healthy controls^3^. Likewise, two studies conducted in North America and Norway indicated a higher prevalence of *F. nucleatum* in stool samples from CRC patients compared to those from healthy subjects^5,11^. Fusobacterium species create a pro-inflammatory environment that facilitates the progression of colon cancer by attracting immune cells to the tumor site^13^. One key mechanism in CRC involving *F. nucleatum* is the virulence factor *FadA*^10,12^. *FadA* plays a crucial role in attaching to and invading host cells, particularly in colon cancer cells, where it triggers signaling pathways that promote oncogene activity and inflammation, leading to increased CRC cell proliferation^14^. Detection of the *FadA* gene can indicate the pathogenicity of the bacterium, and its specificity for *F. nucleatum* makes it a valuable diagnostic target.

Main bacterial identification techniques include culture, nucleic acid probe hybridization, immunohistochemistry, and polymerase chain reaction (PCR)^15,16^. Culture-based methods are considered the gold standard for bacterial identification. However, conventional cultures do not support the anaerobic growth conditions required by *F. nucleatum*, resulting in poor growth^17^. Nucleic acid amplification techniques are highly valuable in life science research. PCR-based amplification is extensively employed for disease diagnosis and has unveiled the prevalence of *F. nucleatum* infection in CRC. Despite its simplicity, accuracy, and ability to discriminate and quantify, PCR is susceptible to inhibitors present in raw biological fluids such as hemoglobin, bilirubin salts, lipids, polysaccharides, phenols, calcium salts, urea, etc.^18–21^. The culture method was found to be inadequate for detecting the presence of *F. nucleatum* in samples. To address the challenges posed by conventional bacterial identification methods like culture and PCR, our focus was directed towards utilizing the LAMP method for rapid detection of *F. nucleatum*. A LAMP test targeting the *FadA* gene was developed^22^. LAMP offers advantages over PCR, such as speed, specificity, and resistance to inhibitors present in biological samples, making it a promising tool for rapid *F. nucleatum* detection^23–29^.

Introduced in 2000, the LAMP method is a cost-effective nucleic acid amplification technique known for its high sensitivity and specificity^30,31^. By utilizing *Bst* DNA polymerase, which is resistant to common PCR inhibitors, LAMP can provide results in a shorter timeframe under isothermal conditions compared to traditional PCR methods^30^. A recent study showed that the *FadA* sequence is specific for *F. nucleatum*, so we used it in our LAMP test. *FadA* primer specificity and sensitivity were confirmed, and the LAMP assay was compared with conventional PCR to detect *FadA* in 57 clinical stool samples^22^. This method, known for its high specificity in pathogen testing, has been widely utilized in clinical settings for various infectious diseases^32^. To the best of our knowledge, LAMP has not yet been used for the clinical diagnosis of *F. nucleatum*.

In this study, a novel modification of the LAMP method called QLAMP-PhR was developed for the quantitative colorimetric detection of *F. nucleatum* using the *FadA* gene as a target^33,34^. This method aims to enable early detection of CRC by quantifying *F. nucleatum* abundance in a single stool sample from the patient with simple equipment and minimal costs. The QLAMP-PhR method is designed to be user-friendly and suitable for point-of-care applications, particularly beneficial in low-resource settings, offering a comparable or even superior alternative to qPCR for monitoring CRC progression.

## Materials and methods

In total, 251 stool samples were used for the LAMP assays. Two hundred stool samples were obtained from 124 male and 76 female CRC patients, and 51 stool samples were obtained from 34 male and 17 female control volunteers who self-reported no evidence of gastrointestinal complaint at the time of sampling were age/sex matched to the CRC patients. All patients were aged 29–79 years. All inclusion and exclusion criteria for CRC patients and control participants are included in Table 1. For comparison, stool samples from CRC patients and healthy controls were investigated for molecular evidence of *F. nucleatum* with QLAMP-PhR assays. The acquired samples were transported immediately on ice to the laboratory of Tishreen University Hospital, where they were stored in a freezer at − 50 °C. All QLAMP-PhR tests were done in the microbiology and biomolecular laboratories of Tishreen University Hospital and in the laboratory sections of the department of molecular biology and biotechnology, Atomic Energy Commission of Syria (AECS). This study was reviewed and approved by the Tishreen University and Tishreen University Hospital Human Research Bioethics Board and Academic Science Research Committee. All of the volunteers provided written informed consent for their participation in this study and the use of their samples.

**Table 1:** Inclusion and exclusion criteria for CRC and control participants.

	CRC patients	Healthy controls
Inclusion criteria	**•** Accepted in Tishreen Hospital-chemotherapy center **•** Individuals diagnosed with CRC between 11/2019 and 11/2020 **•** Confirmation of the diagnosis through pathology reports **•** Any CRC stage or grade **•** Has or hasn’t had chemotherapy or colon surgery	**•** Individuals without a history of colorectal cancer or colon polyps **•** Individuals without a history of any chronic bowel diseases (IBD, crown, IBS, ...) or any Gastrointestinal complaint (diarrhea, jaundice, hemorrhoids, bacterial enteritis, heartburn) for at least 3 weeks prior to the study sampling **•** Similar demographic characteristics to the patient group to minimize confounding factors
Exclusion criteria	**•** Individuals with a history of other cancers to maintain a homogeneous study population **•** Patients with a known genetic predisposition to colorectal cancer **•** Have had pre-sampling radiation therapy **•** Used antibiotics for at least 3 weeks prior to the study sampling	**•** Individuals with a personal history of any cancer to maintain a cancer-free control group **•** Individuals with a family history of colorectal cancer in first-degree relatives **•** Used antibiotics for at least 3 weeks prior to the study sampling **•** Used antacid or PPIs drugs for at least 3 weeks prior to the study sampling

### DNA extraction

Stool samples were removed from the freezer and kept at 37 °C for 20 min prior to use. Genomic DNA was extracted from the stool samples using the RIBO-prep nucleic acid extraction kit (AmpliSens^®^) according to the manufacturer’s instructions (DNA extraction from stool samples). All DNA samples were stored at − 50 °C until used as templates in the LAMP and PCR assays.

### Primer design

The *FadA* gene was previously identified as a sequence enabling specific detection of *F. nucleatum*^35^. BLAST analysis of this sequence (accession no. DQ012971.1) in GenBank confirmed that it exists solely in *Fusobacterium* spp. Three sets of LAMP primers were designed for *FadA* based on GenBank accession number DQ012971.1. Primer Explorer V5 software (https://primerexplorer.jp/e/) was used to design 3 sets of primers. Each set has the outer forward primer (F3), outer backward primer (B3), forward inner primer (FIP), backward inner primer (BIP), forward loop primer (LF) and backward loop primer (LB). Primer sequences are detailed in Table 2. Loop primers (LF, LB) were used to accelerate the amplification reaction. The FIP and BIP primers were linked by a two-thymidine spacer (TT). The first set of primers was chosen and synthesized by the Atomic Energy Commission of Syria (AECS) in Damascus.

**Table 2:** LAMP Primers sets design by Primer Explorer V5 software, the first LAMP Primers set were chosen and synthesized by Atomic Energy Commission of Syria (AECS) in Damascus.

ID:1	Dimer (minimum) dG = -1.68							
Label^a^	5′pos	3′pos	len	Tm^b^	5′dG	3′dG	GC rate^c^	Sequence (5′–3′)^d^
LAMP primers set 1								
F3	36	53	18	57.48	− 5.26	− 5.58	0.50	T T C T G C T T C A G C A T T C G C
B3	213	233	21	56.32	− 4.24	− 4.15	0.38	A G T C T T T G A G C T C T T T G A G A T
FIP			44					T T G C T A A G T T T T G G T A T T C A G C A T C T T A G C A A A T G A T G C A G C A A G T
BIP			45					A A G C A A G A T T C A A T G A A G A A A G A G C T T T G T T C A T T T T G T G C T A G T G C
F2	54	72	19	57.74	− 4.91	− 5.40	0.42	A G C A A A T G A T G C A G C A A G T
F1c	97	121	25	60.01	− 4.49	− 5.14	0.36	T T G C T A A G T T T T G G T A T T C A G C A T C
B2	178	197	20	56.25	− 4.58	− 4.98	0.40	T G T T C A T T T T G T G C T A G T G C
B1c	131	155	25	60.24	− 4.91	− 5.09	0.36	A A G C A A G A T T C A A T G A A G A A A G A G C

ID:11	Dimer (minimum) dG = − 2.09							
Label	5′pos	3′pos	len	Tm	5′dG	3′dG	GC rate	Sequence (5′–3′)
LAMP primers set 2								
F3	65	85	21	55.13	− 5.41	− 4.06	0.38	C A G C A A G T T T A G T A G G T G A A T
B3	264	285	22	55.68	− 4.65	− 4.15	0.36	A G C T A G A T C T T G G T A T T G A G A T
FIP			48					G T C A G C T T G T G C T C T T T C T T C A T T G – G A T G C T G A A T A C C A A A A C T T A G C
BIP			40					G C T G C T A G A C A A G C A C T A G C A – A G C T T C A G C T T G A A G T C T T
F2	97	119	23	57.70	− 5.14	− 4.42	0.39	G A T G C T G A A T A C C A A A A C T T A G C
F1c	141	165	25	62.59	− 5.75	− 4.07	0.44	G T C A G C T T G T G C T C T T T C T T C A T T G
B2	228	246	19	55.99	− 5.09	− 4.24	0.42	A G C T T C A G C T T G A A G T C T T
B1c	166	186	21	62.01	− 6.48	− 4.82	0.52	G C T G C T A G A C A A G C A C T A G C A

ID:10	Dimer (minimum) dG = − 2.40							
Label	5'pos	3'pos	len	Tm	5'dG	3'dG	GC rate	Sequence (5′–3′)
LAMP primers set 3								
F3	36	53	18	57.48	− 5.26	− 5.58	0.50	T T C T G C T T C A G C A T T C G C
B3	228	246	19	55.99	− 5.09	− 4.24	0.42	A G C T T C A G C T T G A A G T C T T
FIP			44					T G A A T C T T G C T T C T T C T T G A T T T G C – G C A G C A A G T T T A G T A G G T G
BIP			44					A G A A A G A G C A C A A G C T G A C G C—A G C T C T T T G A G A T A A T T C A T T G T
F2	64	82	19	55.09	− 6.65	− 4.58	0.47	G C A G C A A G T T T A G T A G G T G
F1c	118	142	25	60.45	− 3.92	− 4.56	0.36	T G A A T C T T G C T T C T T C T T G A T T T G C
B2	203	225	23	55.79	− 5.32	− 4.21	0.30	A G C T C T T T G A G A T A A T T C A T T G T
B1c	147	167	21	63.00	− 3.85	− 6.59	0.52	A G A A A G A G C A C A A G C T G A C G C

^a^Outer forward primer (F3), outer backward primer (B3), forward inner primer (FIP), backward inner primer (BIP), forward loop primer (LF), backward loop primer (LB).

^b^Tm for each zone is designed to be approximately 65 °C (64–66 °C) for F1c and B1c, approximately 60 °C (59–61 °C) for F2, B2, F3 and B3, and about 65 °C (64–66 °C) for the primer ring.

^c^Primers are designed to have a GC content between 40 and 65%.

^d^A primer set contains 4 primers, FIP, F3, BIP and B3. FIP (BIP) consists of a sequence of regions F1c (B1c) and F2 (B2). F1, F2, F3 are approximately 20 bp sequences selected from the target gene, B1, B2, B3 are approximately 20 bp sequences selected from the complement.F1c and F1, B1 and B1c are complementary regions. Once a standard set of LAMP primers (FIP, BIP, F3 and B3) has been identified, loop primers can be designed, which reduces amplification time and improves specificity. Loop primer is designed based on the primer info file of the regular primers suite.

### LAMP reaction

A 25 µl reaction volume was used for the LAMP technique with final concentrations of 2 mM Tris/HCl (pH 8.8), 10 mM KCl, 10 mM (NH4)2SO4, 0.1% Tween 20, 0.8 M betaine, 8 mM MgSO_4_, 1.4 mM each dNTP and 8 U *Bst* DNA polymerase (Jena Bioscience) and phenol red 15 µg/µl as a pH quantitative colorimetric indicator.

**Figure 1:**
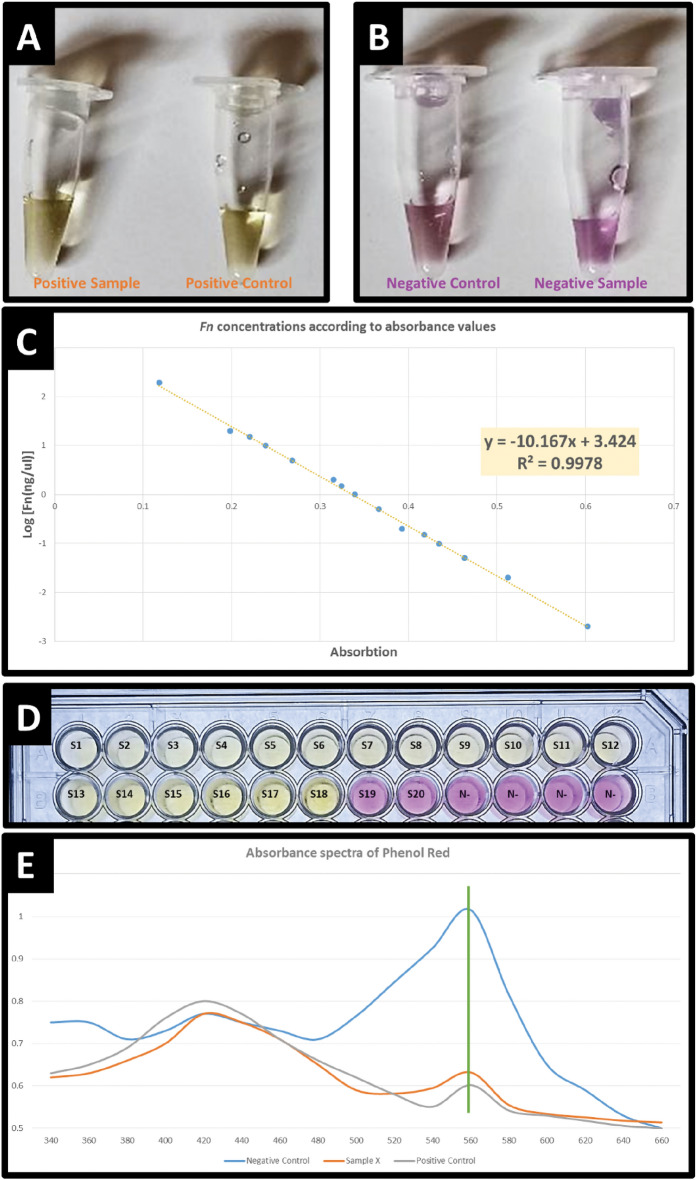
QLAMP-PhR analysis of fadA in *F. nucleatum* with different pH levels of multiple solutions: (**A**) QLAMP-PhR test using phenol red as an indicator, Positive sample and control: phenol red color change from violet-fuchsia to yellow‒orange. (**B**) QLAMP-PhR test using phenol red as an indicator, Negative sample and control: phenol red color (violet—fuchsia). (**C**) By a microplate reader (Thermo Scientific™ Multiskan™ GO Microplate) at 560 nm, we observed a linear relationship between the *F. nucleatum* concentration (*F. nucleatum*. DNA ng/μl) and absorption by function: X = 3.424 − (10.167 × A), X = Log_10_[(*Fn*. DNA ng/μl)/2] → *Fn*. DNA ng/μl = 2 × 10^X^. (**D**) the Serial dilutions of *F. nucleatum* positive standard DNA with QLAMP-PhR test with Absorbance (560 nm), and the LODs of the QLAMP-PhR test is S18 = 0.5 × 10^–3^ ng/μL. (**E**) Absorbance spectra of phenol red at different pH levels of 3 random solutions. Blue is a random negative control, red is a random sample and green is a random positive control. The wavelengths ranged from 420 to 680 nm for visible light spectrum wavelengths.

For each reaction, 40 p.mol FIP and BIP, 20 p.mol LB or LF and 5 p.mol F3 and B3 were used, and 1 µl DNA template (test tube), 1 µl DNA titrate dilutions (positive control) or 1 µl distilled water was used as a no-template control (negative control) (Fig. 1A,B). The reactions were performed at 63 °C for 60 min and then cooled at room temperature for 6 min. The absorbance of each well was read at 560 nm with a microplate reader (Thermo Scientific™ Multiskan™ GO Microplate in Atomic Energy Commission of Syria (AECS) in Damascus).

### Quantitative colorimetric calibration curve

*Fusobacterium nucleatum subsp. nucleatum* [ATCC 25586] was used as a reference strain, and a standard curve was made using DNA extracted from this [ATCC 25,586] reference strain (Fig. 1C). *F. nucleatum* has a genome size of 2.4 Mb, and a single *F. nucleatum* genome weighs 2.43 fg (2.4 Mb/987 Mb [1 pg of double-stranded DNA] = 0.00243 pg). Therefore, 1 ng of *F. nucleatum* DNA contains approximately 411,523 copies of the genome (1000 pg/0.00243 pg)^36,37^.

**Figure 2:**
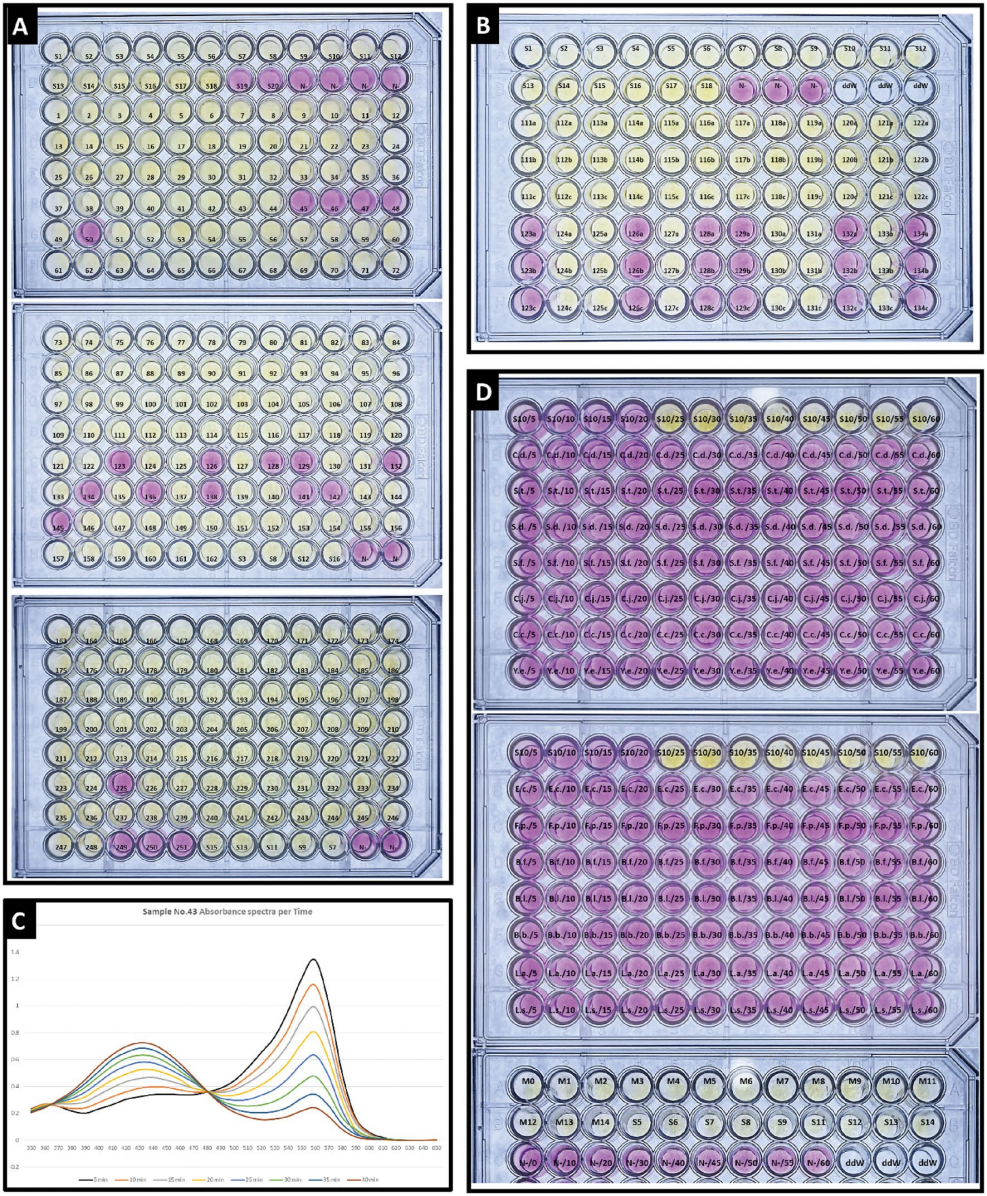
Color photographs highlighting the results obtained at 560nm along with the graph showing the absorbance values over time: (**A**) Color photographs of QLAMP-PhR test results at 560nm (96-well plate). (**B**) A set of images depicting the colored QLAMP-PhR reaction for a group of stool samples (96-well plate). (**C**) The absorption spectrum of sample number 43 in the range of 350nm to 650nm during the QLAMP-PhR test at 5-min intervals. (**D**) Color photographs of Real time—QLAMP-PhR specificity test at 560nm including *F. nucleatum* positive standard DNA and 14 DNA of other most common organisms in stool (*Escherichia coli (E. coli), Faecalibacterium prausnitzii (F. prausnitzii), Bacteroides fragilis (B. fragilis), Bifidobacterium* Spp. *(B. longum, B. bifidum), Lactobacillus* Spp. *(L. acidophilus, L. salivarius), Clostridium difficile (C. difficile), Salmonella typhi (S. typhi), Shigella* Spp. *(S. dysenteriae and S. flexneri), Campylobacter* Spp. *(C. jejuni, C. coli) and Yersinia enterocolitica*).

The quantitative LAMP reaction was carried out with a serially diluted copy number of genomic DNA templates as a control sample colored with phenol red (Fig. 1D). We measured the absorbance spectra of the QLAMP-PhR reaction solutions containing various concentrations of *FadA* DNA amplified based on H^+^ ions when phenol red was used as the colorimetric indicator (Fig. 1E). Samples were considered positive if phenol red color change (from violet/fuchsia to yellow/orange) with amplified *F. nucleatum* DNA was observed (Fig. 2A,B).

In the absence of H^+^ ions, solutions containing phenol red exhibited an absorbance peak at 560 nm, and the absorbance values decreased as the H^+^ ion concentration increased. The absorbance of solutions containing both phenol red and H^+^ ions was also measured at different absorbance wavelengths ranging from 350 to 650 nm at 10 nm intervals (Fig. 1E, 2C). The *F. nucleatum* DNA concentration was estimated by measuring the absorbance at 560 nm and plotting the calibration curve (absorbance against concentration).

Correlating the absorption value and the logarithm of DNA concentrations, a linear regression plot was conducted, producing the standard quantitative analysis curve. Then, a digital value of the *F. nucleatum* DNA copy number amount in each sample was obtained (Fig. 1C).

### Identification of the QLAMP-PhR reaction

Preliminary experiments were revealed no significant primer dimer or self-amplifying reaction.

### QLAMP-PhR sensitivity test

The limit of detection (LOD) was determined for QLAMP-PhR test. Serial dilutions of *F. nucleatum* positive standard DNA were prepared (S1-S20) from 2 × 10^3^ ng/μl to 2 × 10^–5^ copies/μl and measured for QLAMP-PhR. At least three independent experiments of each concentration were assayed, and Double distilled water (ddH_2_O) served as the negative control.

### QLAMP-PhR specificity test

The specificity for *F. nucleatum* of QLAMP-PhR test was evaluated using *F. nucleatum* positive standard DNA and 14 DNA of other most common organisms in stool (*Escherichia coli (E. coli), Faecalibacterium prausnitzii (F. prausnitzii), Bacteroides fragilis (B. fragilis), Bifidobacterium* Spp. *(B. longum, B. bifidum), Lactobacillus* Spp. *(L. acidophilus, L. salivarius), Clostridium difficile (C. difficile), Salmonella typhi (S. typhi), Shigella* Spp. *(S. dysenteriae and S. flexneri), Campylobacter* Spp. *(C. jejuni, C. coli) and Yersinia enterocolitica*). Three independent experiments for each sample were tested, and ddH_2_O served as the negative control. (Fig. 2D).

## Results

We performed the QLAMP-PhR test on 251 stool samples from study participants after DNA extraction. Subsequently, we compared the measured absorbance using a spectrophotometer (Thermo Scientific™ Multiskan™ GO Microplate in the Atomic Energy Commission of Syria (AECS) in Damascus) with the absorbance readings of the DNA standard series for the *Fusobacterium nucleatum* subsp. *nucleatum* [ATCC 25586]. This yielded a precise quantitative value for enumerating *F. nucleatum* in each of the 251 samples.

**Figure 3:**
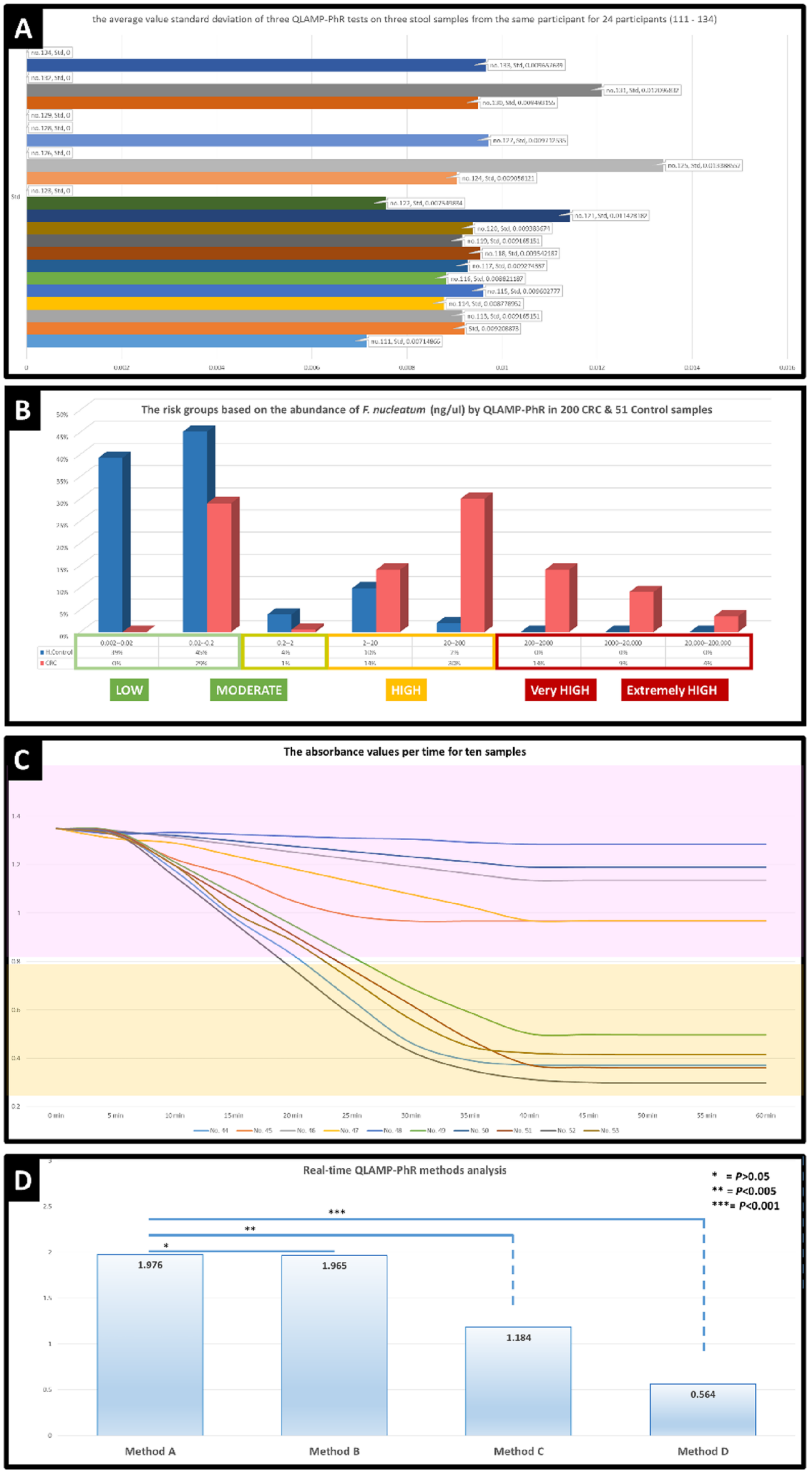
Scientific analysis data graphs of QLAMP-PhR assay results: (**A**) standard deviation of the average value of three QLAMP-PhR tests on three stool samples from the same participant (for 24 participants, no. 111 to no. 134). (**B**) By the QLAMP method, there was evidence for differences in the abundance of *F. nucleatum* in 200 CRC patients (*Fn*. DNA mean 1902 ng/μl) and 51 healthy groups (*Fn*. DNA mean 1.76 ng/μl) (*P* < 0.001). The 8 ranges of *F. nucleatum* DNA ng/μl are from 2 × 10^–3^ to 2 × 10^3^ ng/Μl. (**C**) A graph showing the absorbance values at 560 nm over time (at 5 min intervals) for ten randomly numbered test samples (from No. 44 to No. 53). (**D**) Real-time QLAMP-PhR methods analysis. It shows a significant difference between methods A and C, as well as between methods A and D. However, the results of methods A and B were completely similar, with no significant statistical difference.

On another note, we randomly selected 24 CRC patients (numbered from 111 to 134). Each individual provided three stool samples with a time interval of at least 48 h between each sample. After DNA extraction, the QLAMP-PhR test was conducted. The results for each sample were calculated, and the average of the three results for each participant was considered the final count of the *F. nucleatum* in each sample. We calculated the standard deviation (std) of the *F. nucleatum* values from the three stool samples obtained through the QLAMP-PhR test, which represent three samples from the same individual (Fig. 3A). We found that the standard deviation values for all participants (24 individuals) were significantly close to zero (std ≤ 0.01), as documented in (Excel file, sheet 4). This indicates that the three samples were very consistent and similar, with no significant change in *F. nucleatum* values over approximately one week. Performing the QLAMP-PhR test on just one sample would be sufficient and representative of the other samples from the same individual. This reduces the cost of repeated testing and spares the patient from providing multiple stool samples to assess the presence and abundance of *F. nucleatum*. Taking a single sample from the patient would accurately represent the content of the colon microbiome in terms of *F. nucleatum*.

We were able to classify participants in the study, both CRC patients and healthy individuals, accurately and quantitatively into risk groups (extremely high, very high, high, low, or no risk) based on the abundance of *F. nucleatum* in the stool samples studied using the QLAMP-PhR test. In (Fig. 3B), it was found that over 70% of CRC patients were classified in the risk groups (*F. nucleatum* DNA > 2 ng/μl), with over 56% in the high-risk groups (*F. nucleatum* DNA > 20 ng/μl), and over 26% in the very high-risk groups (*F. nucleatum* DNA > 200 ng/μl). In comparison, high-risk groups were completely absent in healthy participants, with their proportion not exceeding 12% in the low-risk groups, and the majority of them (over 88%) falling within the normal or no-risk values.

### Identification of the QLAMP-PhR reaction

Preliminary experiments were performed only with primers in the absence of any templates to evaluate the negative control precision and revealed no significant primer dimer or self-amplifying reaction.

### QLAMP-PhR sensitivity test

All QLAMP-PhR Sensitivity tests efficiently detected lower copies of *F. nucleatum* DNA, and the LODs of the QLAMP-PhR test was 0.5 × 10^–3^ ng/μl = 0.5 pg/μl = 206 copy/μl. (Fig. 1D).

### QLAMP-PhR specificity test

All three QLAMP-PhR tests only amplified *F. nucleatum*, with no cross-reaction with any of the other organisms. We observed that all fourteen specificity tests were completely negative, and the absorbance value did not drop below 1.3, providing high specificity for the QLAMP-PhR test. The specificity tests were selected to include most bacterial strains present in the gut microbiota. We included a graph displaying the real-time absorbance spectrum for each of the fourteen specificity tests, which we compared to one of the dilution standards (S10) located in (Excel file, sheet 8). Additionally, we performed cross-mixing tests to assess the behavior of the QLAMP-PhR reaction and its potential for cross-reaction when exposed to the mixed DNA from various bacterial strains. We observed a consistent correlation between the outcomes of the fourteen privacy tests and the mixing results, with no statistically significant differences (*P* > 0.9), as documented in (Excel file, sheet 8).

### Modification of the LAMP test by adding phenol red

The addition of phenol red at the end of the reaction is a critical step in making the LAMP test quantitative and capable of providing a numerical value for the *F. nucleatum* count in each individual. It is also important to terminate the test for all tubes simultaneously (60 min in our study) by raising the temperature to 80 °C for 5–10 min to deactivate the *Bst* enzyme.

Hydrogen ions (H^+^) are produced during the polymerization reaction. As the LAMP reaction progresses, *Bst* polymerase incorporates dNTPs (deoxynucleoside triphosphates) onto the target DNA template, resulting in the release of pyrophosphate ions and hydrogen ions. We can rely on the amount of hydrogen ions released from the LAMP reaction in QLAMP-PhR to obtain a quantitative indication of the reaction's progress. This is the principle behind the quantitative calculation of QLAMP-PhR testing. Upon adding a pH indicator, the color changes depending on the amount of hydrogen ions released. By comparing the spectrophotometry of a set of known concentration solutions (serial dilutions of *F. nucleatum* positive standard DNA) with the absorbance of unknown concentration solutions, we can accurately calculate the unknown concentration of samples from patients.

### Real-time QLAMP-PhR analysis

In addition to the QLAMP-PhR method used in this study (method A), we performed three real-time QLAMP-PhR methods (B, C, D) that differed from method (A) in reaction termination timing and addition of phenol red. This was done on ten random samples (no. 44 to no. 53) to investigate the feasibility of implementing real-time QLAMP-PhR methods, monitoring the polymerase reaction and assess their efficacy compared to the QLAMP-PhR method used in this study. In method (B), ten tubes were prepared for each sample, and the testing was initiated simultaneously with the same timing. The reaction was then stopped (by raising the temperature to 80 °C for 10 min) at specific time intervals (e.g., 5 min between each tube). For example, the first tube was stopped after 10 min, the second after 15 min, the third after 20 min, and so on. We added phenol red and measured the absorbance at a wavelength of 560 nm, which provided us with a graphical representation of the QLAMP-PhR reaction in real-time (Fig. 3C). Monitoring the QLAMP-PhR test in real-time (method B) provides us with direct results of the polymeration reaction. It is identical to method (A) but provides a semi-real-time monitoring. It can be relied upon to determine variations in the abundance and richness of *F. nucleatum* in each sample. For instance, sample (no. 52) exhibited the highest abundance among all other samples, as it showed a rapid change in color and absorbance. Sample (no. 51) followed, then sample (no. 44), and so on, until samples (no. 48) and (no. 50), which showed minimal changes in optical absorbance at 560 nm and were considered free of *F. nucleatum*, as documented in (Excel file, sheet 2).

Despite the valuable benefits offered by real-time QLAMP-PhR (method B), such as high accuracy and immediate quantitative results, it requires the preparation of repetitions from the same sample, which can reach up to 10 repetitions per test tube for each sample, with each repetition stopped at a specific time point. This process should also be synchronized with the stopping of the test for each tube in the calibration series used for comparison. Thus, we need ten repetitions for each concentration in the calibration series, (Excel file, sheet 5). All of this requires additional effort, equipment, and financial costs, which would increase the errors in the accompanying readings. These repetitions can be eliminated by adding phenol red directly at the beginning of the reaction and continuously monitoring the color change from reddish–purple to yellow–orange (method C). This would provide an initial idea of the presence of *F. nucleatum* in the sample, as the sample with a higher amount of *F. nucleatum* would exhibit color change first and at a faster rate compared to other samples. However, to obtain accurate quantitative results in real-time, direct measurement of photoabsorbance during the progress of the polymeration reaction is necessary. This requires the presence of a spectrophotometer device along with the reaction mixture (similar to the thermal cycler used in real-time PCR). Thus, additional costs and equipment would be needed, which would compromise one of the main advantages of LAMP, namely simplicity and reduced reliance on complex and costly laboratory equipment and preparations. Moreover, adding phenol red at the beginning of the reaction is not preferable due to its potential negative interference with the reaction and the possibility of providing false results as a foreign substance in the reaction mixture, potentially acting as a contaminant, (Excel file, sheet 5).

Another method, (method D), to perform semi-real-time QLAMP-PhR without preparing repetitions from test tubes for each sample and without adding phenol red at the beginning of the reaction involves withdrawing a small amount of reaction mixture at specific time intervals (e.g., every 5 min), stopping the reaction (by raising the temperature to 80 °C for 10 min), adding phenol red, and measuring the absorbance. However, this method also requires additional effort and tools, and the reaction mixture would be prone to contamination and lack of homogeneity due to the withdrawal of potentially non-homogeneous amounts and the alteration of the reaction mixture. Additionally, this method would require a larger volume of reaction mixture, leading to increased consumption of materials and reagents and, consequently, higher costs. This method would not provide precise real-time monitoring of the reaction but rather intermittent monitoring with time intervals (e.g., every 5 min), (Excel file, sheet 5). We found that this method is not suitable due to its significant deviation from methods A and B, (Fig. 3D), and the presence of false results due to reaction inhibition or loss of materials in the reaction medium.

We believe that measuring the absorbance spectrophotometry of the tube solutions using the QLAMP-PhR method (A) at the end of the test is more important, simpler, and less costly than monitoring the QLAMP-PhR protocol in real-time, (Fig. 3D). Method B requires additional equipment to read the absorbance at time intervals (e.g., every 5 min), which can increase statistical errors by increasing the number of measurements by more than tenfold. Additionally, adding phenol red at the beginning of the test (method C) is not suitable due to its significant deviation from methods A and B, is not preferred as it may negatively affect the QLAMP-PhR protocol, (Fig. 3D). We believe that adding phenol red at the end of the test avoids its negative impact as a potential contaminant that could give false results, like method (C). Adding phenol red to the QLAMP-PhR protocol aims to show a distinct color indicating pH changes in the QLAMP-PhR test, serving as an accurate measure (through its absorbance) for DNA enumeration and quantification in the sample.

### The optimal time to stop the QLAMP-PhR reaction (45 min or 60 min)

Remarkably, the QLAMP-PhR reaction stops after 40–45 min for the ten samples (No. 44 to No. 53), and the absorbance values remain stable despite the ongoing QLAMP-PhR reaction. The statistical graph analysis indicates no significant difference in results among all ten samples after comparing the results at 45 min and 60 min. To confirm this idea, we selected 26 random positive samples (No. 182 to No. 207) and re-applied the QLAMP-PhR reaction, but this time stopping it after only 45 min. We then read the results for each sample and compared them with the previous results obtained when conducting the QLAMP-PhR test for 60 min. Interestingly, we found that the values were either similar or identical across all 26 samples, with no statistically significant differences (*P* > 0.9), as documented in (Excel file, sheet 2). Therefore, it is advantageous to halt the QLAMP-PhR reaction entirely after 45 min to achieve optimal efficiency and accurate results within the shortest timeframe.

### Spectrophotometry results and calculation

A spectrophotometry scan was performed in the visible range (350–650 nm) for several QLAMP–PhR test tubes (random sample tube/negative/positive) to confirm the maximum absorption peak of the phenol red solution. These peaks were found to be located at 530 nm and 590 nm, specifically at 560 nm. Therefore, a wavelength of 560nm was adopted to measure the spectrophotometry of all QLAMP–PhR test tubes of samples in this study.

Additionally, a spectrophotometry scan was conducted in the visible range (350–650 nm) for a random sample (No. 43) that exhibited changes in spectra and absorbance peaks during the reaction of the QLAMP–PhR test over time intervals of 5 min. The (Fig. 2C) demonstrates that the peak at 560 nm will be highly useful in calculating the results by measuring the spectrophotometry for all QLAMP-PhR test tubes of the participating samples at 560 nm.

During the QLAMP-PhR test conducted on the dilution series, we observed that the results followed a decimal logarithmic curve. Hence, the decimal logarithm of the DNA quantity for *F. nucleatum* per microliter of stool was adopted for comparison with the results. The dilution series of the Fusobacterium nucleatum subsp. nucleatum strain [ATCC 25586] served as the reference scale for our study and for comparing all QLAMP-PhR test results of the participating samples.

The spectrophotometry was measured using the spectrophotometer (Thermo Scientific™ Multiskan™ GO Microplate in the Atomic Energy Commission of Syria (AECS) in Damascus) for all samples at a wavelength of 560nm, and a linear relationship was obtained for the decimal logarithm of the DNA copy number for *F. nucleatum*. All measurements and comparisons were conducted based on this concept (*F. nucleatum* DNA ng/μl) (the quantity of *F. nucleatum* DNA per microliter of stool).

$$\begin{aligned} {\text{X }} & = {\text{ Log}}_{10} \left[ {\left( {Fn.\,{\text{DNA}}\,{\text{ ng}}/{\text{ul}}} \right)/2} \right] \to Fn.\,{\text{DNA}}\,{\text{ ng}}/{\text{ul }} \\ & = \, 2 \, \times \, 10^{{\text{X}}} , \, 1\,{\text{ ng}}/{\text{ul }} = 411,523 \, \,Fn. \, \,{\text{DNA }}\,{\text{copies}}/{\text{ul}} \\ \end{aligned}$$

Through the QLAMP-PhR test on the dilution series, we found that the last dilution giving a positive result was at 0.5 × 10^–3^ ng/μl, which represents the minimum detection limit for *F. nucleatum* in our study, equivalent to 206 copies per microliter of stool.

### The QLAMP-PhR reaction and dilution factor (DF)

To verify the accuracy and precision of the work, the authors compared the absorbance values of 15 random positive samples without and after dilution for several repetitions (10×, 100×, 1000×). This comparison relied on the concentration values of *F. nucleatum* (log_10_ ng/μl) after adjusting each sample with its corresponding dilution factor (DF). Our observation indicates that sample dilution (tenfold to a thousandfold) aligns closely with the actual value (after adjusting for the dilution factor) and has no impact on the QLAMP-PhR reaction process with no statistically significant differences (*P* > 0.9), as documented in (Excel file, sheet 6). Consequently, we can dilute the high-concentration sample to achieve an absorbance value within the range of (0.2 to 0.5), which corresponds linearly to the relationship between absorbance and concentration according to the Beer-Lambert law.

### Number of samples for each participant and sample homogeneity

For increased accuracy and to minimize potential errors due to stool sample heterogeneity, we randomly selected 24 CRC patients (numbered from 111 to 134). Each individual provided three stool samples with a time interval of at least 48 h between each sample. QLAMP-PhR testing was performed on the three samples from the same individual, and the average was calculated as shown in the (Excel file, sheet 4). By calculating the difference between the average and the values of the three results for each sample, we found that the difference was significantly close to zero (std < 0.05) in almost all samples. Therefore, we can conclude that taking a single sample from each participant or from each CRC patient would be sufficient to provide a true, homogeneous, and accurate representation of the quantity of *F. nucleatum* in the intestinal environment over a period of approximately one week (due to the interval of at least 48 h between the collection of the three stool samples). The variations in the colonic microbiome may vary and be influenced by the digestive process and food quality. Thus, these three samples would provide a genuine and realistic expression of the presence of *F. nucleatum* in the colon over a period of approximately one week. Additionally, researchers in this field can combine the DNA extracted from the three samples belonging to the same individual, then perform QLAMP-PhR testing on the mixed sample, which will represent the colonic content in the patient's stool over a full week.

### QLAMP-PhR test results

At the end of the QLAMP-PhR tests, we obtained a set of results related to the abundance of *F. nucleatum* in the stool samples. We observed a significant and substantial increase in the CRC patient group compared to the healthy control group. The average values of the results were 1902 ng/μl for the CRC patient group (*F. nucleatum*. DNA mean) and 1.76 ng/μl for the healthy control group (*F. nucleatum*. DNA mean). This difference was statistically significant (*P* < 0.001), as documented in (Excel file, sheet 1).

We can conclude that detecting the abundance of *F. nucleatum* in stool samples provides ease of handling for physicians, patients, and healthcare providers dealing with CRC cases. CRC is considered a chronic and slowly progressing disease, and the treating physician requires additional biological evidence to assess the progression of the disease over time, during treatment, or in cases of relapse. Asking the patient to provide a stool sample to evaluate the presence and abundance of *F. nucleatum* would be an easy and convenient step compared to performing a lower gastrointestinal endoscopy to obtain the required sample for assessing the presence and abundance of *F. nucleatum*. The stool sample provides comprehensive information about the colonic content of *F. nucleatum*, which is part of the intestinal microbiome.

### Ethics approval

Ethical approval has been granted as of October 16, 2019, according to the decision of research registration by Tishreen University Council (No.299/15–10-2019), and Recruitment start as of October 16, 2019. All participants gave their consent to anonymously publish their entered data at Tishreen University Hospital-Latakia-Syria, which was compatible with the Declaration of Helsinki.

### Consent for participation

Informed consent was obtained from all individual participants included in the study.

## Discussion

The principle of LAMP involves self-synthesis of DNA by strand displacement using highly active DNA polymerase with strand shifting activity and a specially designed set of two internal primers and two external primers. Nucleoside tri-phosphates are polymerized into DNA polymers by the action of *Bst* polymerase enzymes that use the complementary strand as a template. Incorporation of the nucleotide into the polymer results in the liberation of a pyrophosphate and a hydrogen ion and the formation of a sugar-phosphate backbone. Phenol red was reported to be useful as a pH colorimetric indicator for the titration of Mg^+2^ and hydrogen ions (H^+^)^38^. Therefore, we hypothesized that it could be a novel indicator for the LAMP reaction by monitoring the change in the Mg^+2^ and H^+^ ions concentration since the large fragment of *Bst* DNA polymerase synthesizes DNA under alkaline conditions (pH 8.8 at 25 °C). The color of phenol red changes depending on the pH of the solution; when the solution contained 8 mM Mg^+2^ ions and no dNTPs, its color was yellow at pH values below 6.2 and fuchsia-violet at pH values over 8.2 (Fig. 1A,B). After the addition of 1.4 mM dNTPs to this solution, the color of phenol red changed from yellow to fuchsia-violet irrespective of the pH. By QLAMP-PhR test, This color change is induced with the chelation of Mg^+2^ ions by dNTPs and Increased release or production of (H^+^) with polymeration reaction^39^.

### Stool samples and the pH variation

Previous studies indicate that the LAMP test is not affected by variations in the pH of stool samples^18,19^. The polymerization reaction proceeds smoothly, yielding qualitative results. LAMP testing is considered stable against some PCR inhibitors and can be performed without the DNA extraction step using untreated samples, according to several previous studies^20,40–43^. However, the quantitative QLAMP-PhR protocol primarily relies on the change in phenol red color due to pH variations. In a positive reaction, the color changes to yellow-orange, where the color intensity and absorbance are influenced by any contaminant altering the pH. Therefore, a DNA extraction step is necessary to avoid this issue.

The DNA extraction step is considered essential and crucial in the QLAMP-PhR protocol. While phenol red plays a pivotal role in measuring the colorimetric QLAMP-PhR absorbance of the test solutions according to the acidity resulting from the release of H^+^ ions during the polymerization reaction, it can be affected by impurities altering the pH and yielding false results. However, the presence of the *Bst* polymerase enzyme's buffers will provide the primary safeguard for the pH indicator, phenol red. Phenol red will precisely follow the *Bst* polymerase enzyme's path, regulated by the buffers used in the QLAMP-PhR test.

Study^20,21^ indicated the high resistance of the LAMP method to various inhibitors (hemoglobin, bilirubin salts, lipids, polysaccharides, urea, etc.) in different biological samples, including stool samples. The detection of Salmonella bacteria in raw stool samples was performed without DNA extraction, and all results were qualitatively consistent with the LAMP analysis results after DNA extraction. However, the quantitative results differed due to the influence of inhibitors and impurities. This suggests the possibility of directly applying the LAMP method to raw samples, with the polymerization reaction proceeding reliably. Nevertheless, we recommend applying the QLAMP-PhR protocol to samples after DNA extraction, and currently, it is advised not to skip this step to obtain accurate quantitative results that can be compared precisely and reliably with the calibration series.

### Color change timing and decision-making

By monitoring the real-time QLAMP-PhR reaction of the standard series (S1–S20), we observed that the highest concentration, S1, exhibited the earliest color change, followed by the lower concentrations, as documented in (Statistical Excel file, Sheet 7). The slowest color change and the lowest positive detection limit (LOD) were observed at S18. S1 demonstrated an absorbance change after 15 min from the initiation of the real-time QLAMP-PhR reaction (shifting from 1.35 to below 0.8), whereas S18 exhibited a change after 30 min. Notably, all negative values remained constant at both 45 and 60 min. Therefore, continuing the QLAMP-PhR test beyond 45 min does not alter the reaction outcome, and 45 min suffice for obtaining accurate results in the shortest possible time. All positive standard tubes and most of the positive tubes in our study exhibited a color change between 15 and 30 min from the test initiation. This correlates with the decrease in absorbance below 0.8 after 15 min and the stabilization observed after 30 min. As a result, a qualitative positive reaction can be ascertained within the 15–30 min window, enabling us to reach a definitive positive or negative conclusion within this timeframe.

### The maximum absorbance wavelength of phenol red

By tracking the real-time QLAMP-PhR reaction for sample (no. 43), we observe the presence of two distinct absorbance peaks (Statistical Excel file, Sheet 2). These peaks correspond to the color indicators, which exhibit two primary colors (violet–red and orange–yellow) that vary based on pH levels. We chose the maximum wavelength of 560 nm as it exhibits the most significant difference in absorbance when the test is positive, further enhancing the sensitivity of the QLAMP-PhR method compared to traditional LAMP techniques. We excluded the reading of absorbance values of the samples at the second maximum wavelength of 420 nm, although it could provide excellent results but with slightly less accuracy than at 560 nm. Utilizing dual readings at wavelengths of 560 nm and 420 nm can be a hallmark of the quantitative QLAMP-PhR method compared to traditional LAMP or other PCR methods.

### QLAMP-PhR test to become a point-of-care test (POCT)

In fact, based on the results, we can say that rapid detection of *F. nucleatum* using the QLAMP-PhR test is easily applicable in medical laboratories. This test relies on estimating the concentration of bacteria in a specific volume of stool. Developing the QLAMP-PhR test to become a point-of-care (POC) test will face some challenges that the patient alone may not be able to overcome. Therefore, currently, we can say that the QLAMP-PhR test should be performed in a laboratory, clinic, or any healthcare facility, no matter how basic it is, as it requires simple skills related to volume and weight estimation. However, the results we have obtained will serve as a strong motivation for researchers in this field to further develop this method into a POC test, with the support of specialized industrial companies designing these tools and kits.

The ability to classify CRC patients, healthy individuals, or individuals predisposed to CRC into risk groups based on precise *F. nucleatum* values in the stool would be an extremely important step in healthcare for various individuals. It would provide significant benefits in terms of offering early therapeutic and interventions for those individuals falling within the risk groups associated with *F. nucleatum*. Additionally, this analysis could be included in the required investigative protocols for gastrointestinal diseases, intestinal polyps, and various types of cancer patients. It could offer a genuine and serious attempt to reduce future CRC incidence and lower the level of infection through accurate early detection.

### QLAMP-PhR can classify CRC patients

Some studies have been able to classify individuals based on the abundance of *F. nucleatum* in the colon as either high or low. Meanwhile, other studies have indicated a specific number of *F. nucleatum* per gram of stool sample, which serves as a determinant for the extent of *F. nucleatum* presence^4,5,14,44^. Most of these studies focused on PCR-based detection methods. However, the QLAMP-PhR method has been able to provide an accurate and rapid quantification with extremely simple capabilities and low costs. We can say that the QLAMP-PhR method detects the number of DNA copies/μl of the stool sample and establishes a scale and reference for the degree of abundance and the presence or elevation of *F. nucleatum* in the colon (Fig. 3B).

The ability to classify CRC patients, normal individuals, or individuals predisposed to CRC into risk groups based on precise *F. nucleatum* values in the stool would be an extremely important step in healthcare for various individuals. It would provide significant benefits in terms of offering early therapeutic and interventions for those individuals falling within the risk groups associated with *F. nucleatum*. Additionally, this analysis could be included in the required investigative protocols for gastrointestinal diseases, intestinal polyps, and various types of cancer patients. It could offer a genuine and serious attempt to reduce future CRC incidence and lower the level of infection through accurate early detection.

by applying the quantitative QLAMP-PhR test, we can obtain an accurate biological marker for the extent of colon colonization by opportunistic inflammatory gut microbiota *F. nucleatum*^45^, and improve diagnostic efficiency^46,47^. The treating physician or medical supervisor can easily and regularly request this analysis at a low cost to monitor the progression of CRC or other inflammatory gastrointestinal diseases over time. This analysis would be accessible to any laboratory with simple capabilities and equipment, achieving continuous monitoring at a low cost. It would provide the treating physician with various therapeutic options by periodically monitoring *F. nucleatum* abundance to assess the effect of treatment on the availability of these bacteria in the stool, which reflects the colonic microbial environment.

Furthermore, we also found that a single stool sample is sufficient to provide accurate information about the abundance of *F. nucleatum* microbiota in the colon over approximately one week.

### QLAMP-PhR method compares discussion with commonly employed methods

The development of a QLAMP-PhR method with phenol red has introduced a novel approach to real-time monitoring of DNA amplification. This method offers distinct advantages over non-quantitative Lamp methods or PCR methods. By the QLAMP-PhR novel protocol we can discuss the differences and benefits of the quantitative Lamp method with phenol red compared to non-quantitative Lamp methods or PCR with 4 points (Principle and Process, Real-time Monitoring, and Sensitivity and Specificity):

1. Principle and Process: The non-QLamp method is based on the isothermal amplification of target DNA using a set of primers and a DNA polymerase with strand displacement activity. However, it lacks the ability to provide quantification of the amplification process. PCR involves the amplification of target DNA using specific primers and a DNA polymerase in a cyclic manner, including denaturation, annealing, and extension steps. Real-time PCR allows for the measurement of DNA amplification during the reaction, enabling quantification. The QLAMP-PhR method incorporates a pH indicator, phenol red, into the reaction mixture. As the amplification proceeds, the release of protons due to DNA synthesis causes a color change in the reaction mixture, which can be monitored in real-time.
2. Real-time Monitoring: The non-QLamp method relies on visual inspection or endpoint detection methods such as gel electrophoresis or turbidity measurements to determine the presence or absence of amplification. It does not provide real-time monitoring or quantification of the amplification process. Real-time PCR enables the continuous monitoring of DNA amplification using fluorescent probes or intercalating dyes. This allows for the quantification of the target DNA based on the cycle threshold (Ct) value. The QLAMP-PhR method can provide real-time monitoring of the amplification process through the color change of the reaction mixture. The intensity of the color change correlates with the amount of target DNA present, allowing for quantitative analysis.
3. Sensitivity: Both the non-quantitative LAMP and QLAMP-PhR methods have demonstrated high sensitivity for detecting the target nucleic acid. The non-quantitative LAMP method is known for its robust amplification and can detect low amounts of the target sequence, often comparable to or even better than PCR. Similarly, QLAMP-PhR, being a quantitative colorimetric version of LAMP, offers high sensitivity for target detection and allows for quantification of the initial target concentration. Scientific data from real-time QLAMP-PhR tests reveal a diverse range of outcomes for both negative and positive samples. This is achieved through qualitative assessment by monitoring color changes during the reaction in positive samples (violet-red to orange-yellow). Specifically, in our assessment of the LAMP qualitative test's sensitivity, we found that S18 = 0.5 × 10^–3^ ng/μl was the lowest dilution yielding a positive outcome through color change, as documented in (Statistical Excel file, Sheet 7). This value closely aligns with the LOD established by the LAMP method utilizing *FadA* and *nusG* gene primers in Huang et al.^22^. Similarly, when employing the quantitative QLAMP-PhR method, we precisely ascertained the assay's sensitivity by measuring absorbance and defining positive reactions through absorbance values at 560 nm. The quantitative real-time QLAMP-PhR method confers the ability to accurately detect the lowest positive concentration or the minimum amount of DNA required for the QLAMP-PhR reaction in a digital result. Consequently, this approach avoids visual color discrimination errors that may occur in qualitative LAMP methods. It can be stated that the real-time quantitative QLAMP-PhR method achieves high sensitivity in the shortest possible time (40–45 min). In contrast, the PCR method utilizing *FadA* gene primers failed to detect concentrations below 2.25 × 10^–3^ ng/μl of Fn DNA, as per the findings of Huang et al.^22^. This underscores the superior sensitivity of the QLAMP-PhR method, which successfully detects lower quantities of Fn DNA using solely *FadA* gene primers, thereby offering simplicity, flexibility, and cost-effectiveness. Furthermore, through real-time monitoring of the QLAMP-PhR reaction for the standard dilution series (S1–S20), we observed a discernible color shift when the absorbance value transitioned from above 0.8 to below it. Consequently, an absorbance value of 0.8 serves as the approximate threshold for differentiating positive from negative samples in the QLAMP-PhR method.
4. Specificity: During the real-time QLAMP-PhR reaction monitoring for specificity tests (Statistical Excel file, Sheet 7), we did not observe any absorbance changes throughout the duration of the test for the other fourteen bacterial strains, indicating negative results. In contrast, S10 displayed a significant absorbance change consistent with a positive QLAMP-PhR reaction. When performing the cross-mixing specificity tests (M1–M14), all mixing tests perfectly matched the single comparison test (M0), demonstrating the high purity and specificity of the QLAMP-PhR reaction using *FadA* gene primers. These findings align with the results obtained by a Huang et al.^22^ study that employed *FadA* and *nusG* gene primers for *F. nucleatum* strains in LAMP and PCR molecular biology assays.

Both the non-quantitative LAMP and QLAMP-PhR methods provide high specificity in detecting the target sequence. They rely on specific primers designed to bind to the target sequence, ensuring specificity in amplification. The specificity of both methods is critical in reducing the likelihood of false-positive results and ensuring accurate detection. The difference between the two methods lies in the quantification aspect. While non-quantitative LAMP focuses solely on qualitative detection, QLAMP-PhR goes a step further by enabling colorimetric quantification of the target nucleic acid. By incorporating additional steps and controls, QLAMP-PhR allows for the determination of the initial concentration of the target sequence, providing a quantitative measure. It’s important to note that the sensitivity and specificity of both methods can be influenced by various factors, including primer design, reaction conditions, and the specific target sequence being amplified. Optimizing these factors is crucial to achieving the best sensitivity and specificity for a given application. In summary, QLAMP-PhR provides an additional capability of quantitative measurement, allowing for precise quantification of the target nucleic acid concentration.

To the best of our knowledge, This study is the first to report the application of QLAMP-PhR for the detection of *FadA* in *F. nucleatum* since this novel nucleic acid amplification method was developed by whom^48^. The developed QLAMP-PhR colorimetric assays are simple and quick, do not require gel electrophoresis and PCR of the amplified product, and the results can be interpreted with the naked eye. This QLAMP-PhR has several advantages over other nucleic acid amplification techniques: easy operation, no special equipment, low risk of contamination, higher sensitivity and speed as well as the ability to detect high throughput DNA and RNA expression. Therefore, this quantitative colorimetric test is suitable not only for laboratory research but also for the clinical diagnosis of many infectious diseases.

Based on our clinical results, the QLAMP-PhR method emerges as a simple, rapid, and cost-effective approach for quantitatively screening the presence of *F. nucleatum* across all populations.

## Conclusion

The developed QLAMP-PhR detection method is a sensitive, rapid, and efficient approach for detecting the *F. nucleatum* pathogen. This test has potential applications in hospitals, healthcare stations, and other clinical settings. The QLAMP-PhR assay may become a new gold standard for *F. nucleatum* detection.

### Supplementary Information

Supplementary Information.

## Data Availability

All data generated or analysed during this study are included in this published article (Statistical Excel file, 8 Sheets).

## Abbreviations

*F. nucleatum*: *Fusobacterium nucleatum*
CRC: Colorectal cancer
LAMP: Loop-mediated isothermal amplification
QLAMP-PhR: Quantitative LAMP colorimetric-Phenol Red
PCR: Polymerase chain reaction
AECS: Atomic energy commission of Syria

## LAMP primers

F3: Outer forward primer
B3: Outer backward primer
FIP: Forward inner primer
BIP: Backward inner primer
LF: Forward loop primer
LB: Backward loop primer
